# The potential yield of geographically targeted tuberculosis contact investigation in urban Uganda

**DOI:** 10.1017/S0950268825100605

**Published:** 2025-09-26

**Authors:** Katherine O. Robsky, Annet Nalutaaya, Peter James Kitonsa, James Mukiibi, David Isooba, Olga Nakasolya, Emily A. Kendall, Jonathan Zelner, Jennifer M. Ross, Achilles Katamba, David W. Dowdy

**Affiliations:** 1 Center for Global Health Practice and Impact, Georgetown University, Washington, DC, USA; 2 Uganda Tuberculosis Implementation Research Consortium, Walimu, Kampala, Uganda; 3 Division of Infectious Diseases, Johns Hopkins University School of Medicine, Baltimore, MD, USA; 4 School of Public Health, Department of Epidemiology, University of Michigan, Ann Arbor, MI, USA; 5 School of Public Health, Center for Social Epidemiology and Population Health, University of Michigan, Ann Arbor, MI, USA; 6 Division of Allergy and Infectious Diseases, Department of Medicine, University of Washington, Seattle, WA, USA; 7 Clinical Epidemiology and Biostatistics Unit, Department of Medicine, Makerere University, College of Health Sciences, Kampala, Uganda; 8 Johns Hopkins Bloomberg School of Public Health, Department of Epidemiology, Baltimore, MD, USA

**Keywords:** tuberculosis, active case finding, contact investigation, global health, geographic information systems, epidemiology

## Abstract

We investigated the potential yield of conducting active case finding for tuberculosis (TB) within a defined geographic radius (50 or 100 m) around the households of individuals diagnosed with TB at health facilities. In a well-defined geographic area within Kampala, Uganda, residential locations were determined for 85 people diagnosed with TB at local health facilities over an 18-month period and for 60 individuals diagnosed with TB during a subsequent community-wide door-to-door screening campaign. Ten of the individuals diagnosed through community screening lived within 50 m of an individual previously diagnosed with TB in a local health facility (TB prevalence: 0.98%), and 15 lived at a distance of 50–100 m (prevalence: 0.87%). The prevalence ratio was 1.4 (95% confidence interval (CI): 0.7–2.9) for those <50 m and 1.2 (95% CI 0.6–2.2) for those 50–100 m, compared to >100 m. Using TB notifications to identify areas for geographically targeted case finding is at most moderately more efficient than screening the general population in the context of urban Uganda.

## Background

Increasing tuberculosis (TB) case detection is a pillar of the End TB Strategy, but to be efficient, case finding must target populations at increased risk for TB [1]. While screening among household contacts of people diagnosed with TB is a high-yield activity [2], the majority of TB transmission in high-burden settings occurs outside the household. Therefore, finding other efficient approaches to TB case finding is essential to achieve broader population level impact [3]. Studies have suggested that a large percentage of households that either are direct neighbours of or lie within 50 m of ‘index’ households may include individuals with undiagnosed TB [4, 5]. Using data from a study of TB prevalence and transmission in a small, densely populated area of Kampala, Uganda (STOMP-TB) [6], we aimed to investigate the potential yield of active case finding conducted within a defined geographic radius (50 or 100 m) around the homes of individuals who were diagnosed with TB at local health facilities.

## Methods

### Study design

The parent study enrolled adults (>15 years) who lived within the boundaries of the study area and were diagnosed with TB at any of four local health facilities (‘facility-diagnosed individuals’), from May 2018 to November 2019. Facility-based TB diagnosis was at the discretion of local clinicians, with Xpert MTB/RIF (‘Xpert’, Cepheid, Inc., Sunnyvale, CA, USA) generally used for confirmation. Consenting participants diagnosed with TB were asked to describe their location of residence using street names and landmarks; given the informal housing arrangements common in this area, street addresses were not considered reliable. Study staff used this information to geocode a location on Google maps and verified this location with each participant.

From February to November 2019, the study team systematically went door to door, offering testing for TB using Xpert MTB/RIF Ultra (‘Xpert Ultra’, Cepheid Inc., Sunnyvale, CA, USA) to all adult residents of the area, regardless of symptoms. Door-to-door screening was completed as a single wave through each parish within the study area to identify individuals with undiagnosed TB who may not have sought care at one of the local health facilities. Study staff conducted sensitization events with local leaders and health workers in each study region. Screening was conducted during daytime hours (9 am–5 pm); in the event that participants were not home, study staff would return up to two more times in attempt to reach all adult residents of the study area. Study staff captured global positioning system (GPS) coordinates at each consenting household. We included all participants with positive Xpert Ultra results (including trace) as having prevalent TB. Household contact investigations, with referral for TB preventative therapy per the Uganda National guidelines, were conducted separately from the door-to-door screening. Further details of the parent study, including facility-based TB diagnosis and community-based activities including door-to-door case finding, event-based screening, and household contact investigation are described elsewhere [7].

### Statistical analysis

We estimated the prevalence of TB among door-to-door screening participants (excluding household contacts, who were identified separately via contact investigation) within potential screening radii of 50 and 100 m around the household location of each facility-diagnosed individual. Individuals with prevalent TB were included only once; in the event that a door-to-door screening participant lived within 100 m of multiple facility-diagnosed individuals with TB, we used the closest. We then calculated the prevalence ratio, comparing TB prevalence within these screening radii to TB prevalence in areas >100 m away from all individuals with facility-diagnosed TB. We compared sociodemographic characteristics of individuals with prevalent TB living within 100 m of an individual with facility-diagnosed TB to those living >100 m away using Fisher’s exact and Wilcoxon rank sum tests.

### Ethical considerations

The study was approved by the Johns Hopkins Bloomberg School of Public Health Institutional Review Board (IRB Number 11353) and the Higher Degrees, Research and Ethics Committee of the Makerere University School of Public Health, Kampala-Uganda (Study Protocol Number 544). All participants provided written informed consent (or written assent and parental consent for those 15–17 years old) for all study activities.

## Results

Eighty-five people from the study area were diagnosed with TB at health facilities during the study period; six were excluded due to missing or out of study area GPS coordinates. The majority of individuals with facility-diagnosed TB had bacteriologic confirmation of TB (69/85, 81%); the remaining were diagnosed clinically by the healthcare provider. Of 34135 adults in the study area, 8189 were home and agreed to participate in door-to-door screening; additional details on the results of the case-finding activities are described elsewhere [7]. Valid Xpert Ultra results were obtained from 7992/8189 (98%) individuals in 5803 households (5126 with GPS coordinates within the study area). There were 60 people diagnosed with TB via door-to-door screening (seven excluded due to missing GPS coordinates), two of whom shared a household (>100 m from the nearest household of a facility-diagnosed individual).

**Figure 1.**
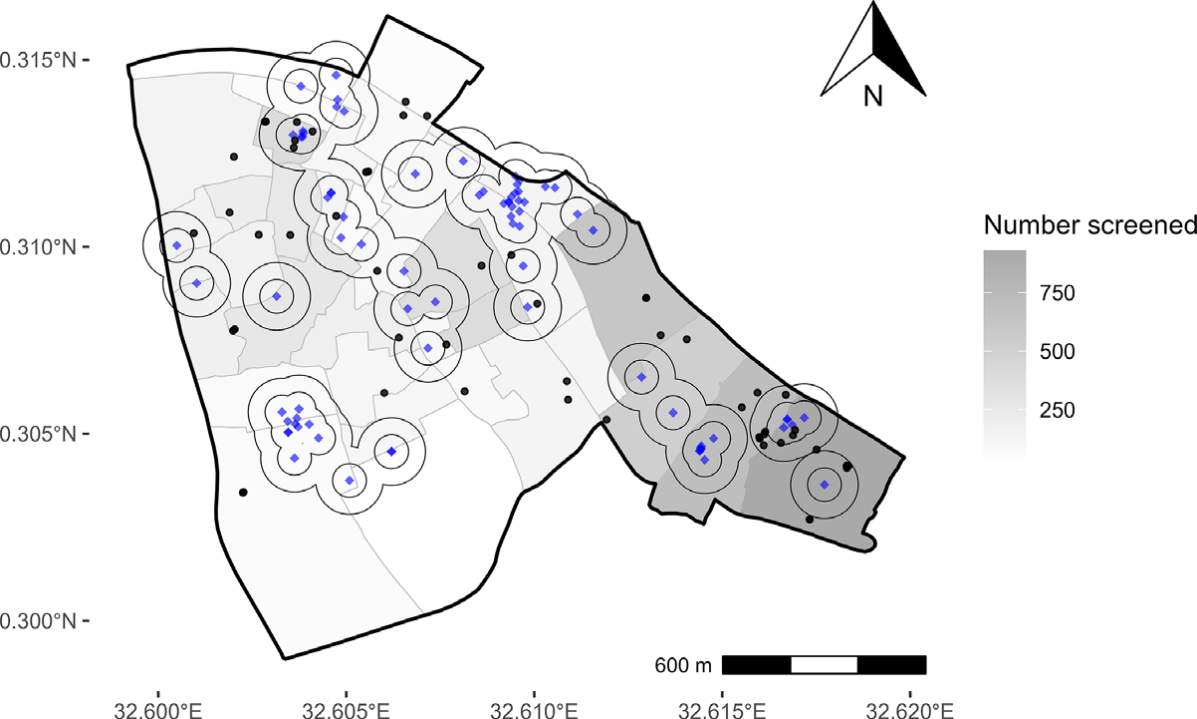
Potential yield of screening for TB within 50 and 100m of index households. Blue dots indicate the locations of residence for individuals diagnosed with TB in health facilities, and black dots indicate residences of individuals who had positive Xpert results during door-to-door screening. The circles represent areas within 50 and 100m radii from each person diagnosed with TB at a health facility. Dots inside the screening radii indicate individuals with TB who could be detected by geographically targeted screening within that radius. The number of individuals who completed community-based screening in each administrative zone is indicated by the shading (darker colors indicate a larger number of individuals screened).

Of the 1016 individuals (14% of all community-based testing participants) who lived within 50 m of an individual with facility-diagnosed TB and completed Xpert Ultra testing, 10 had positive test results (prevalence = 0.98%) (Figure 1). Of the additional 1829 individuals (23% of all community-based testing participants) who lived between 50 m and 100 m from an individual with facility-diagnosed TB and who completed Xpert Ultra testing, 15 had positive results (prevalence = 0.82%). By comparison, the Xpert Ultra-positive prevalence among those living >100 m from an individual with facility-diagnosed TB was 0.68% (35/5147). Thus, screening all people living within 50 m of individuals with facility-diagnosed TB would require testing 14% of community members and would detect 19% of individuals with undiagnosed TB in the community; expanding screening to 100 m would require testing an additional 23% of the population to detect an additional 28% of individuals with undiagnosed TB. Compared with people living >100 m from any person diagnosed with TB in study facilities, the prevalence ratio among those living within 50 m was 1.4 (95% confidence interval (CI) 0.69–2.9), and among those living 50–100 m away was 1.2 (95% CI 0.63–2.2).

Individuals with undiagnosed TB living within 100 m of individuals with facility-diagnosed TB were not significantly different than their counterparts living more than 100 m away according to age (median 31 vs. 29 years), sex (68% vs. 61% female), self-reported history of incarceration (16% vs. 18%), or self-reported recent contact with a person with TB (32% vs. 32%). They did have a higher prevalence of HIV (32% vs. 11%, *p* = 0.090) and report lower monthly household income (300000 vs. 500000 Ugandan Shillings (approximately $82 vs. $134), *p* = 0.11) and higher frequency of ever having a TB diagnosis in their household (36% vs. 25%, *p* = 0.47), though none of these associations were statistically significant.

## Discussion

In this urban Ugandan community, we observed that the prevalence of undiagnosed TB was only marginally higher among households within 50 or 100 m of a household with a person with TB diagnosed at a health facility as compared to households more than 100 m from a person with facility-diagnosed TB. While expanding contact investigation to include all households within 50 m of the index household would increase the number of individuals diagnosed with TB, this approach is not significantly more efficient than community-based screening of the general population in this urban Ugandan setting.

There are significant challenges to a door-to-door screening approach, even in high burden settings. Availability of individuals for home-based screening in this area varied throughout the day and week [8], and the individuals most likely to be reached by home-based screening may not be those at highest risk of having undiagnosed TB. For example, more than 60% of individuals diagnosed with TB during home-based screening were women, though the risk of TB is higher in men. The challenge of reaching men during community-based TB screening programmes was noted in our study and others; approaches may need to be modified based on timing and location of screening in order to reach men and other populations at higher risk for TB [8, 9]. There may also be more efficient methods for identifying people with undiagnosed TB in this community; for example, the prevalence of TB in both our public screening events (2.0%) and our household contact investigations (1.9%) was nearly double the prevalence of TB compared to screening those living within 50 m of a household of a facility-diagnosed individual [10]. Further work is needed to develop data-driven approaches for targeting TB screening interventions to high-risk groups, such as optimizing hotspot-based testing, leveraging workplace screening programmes, or conducting outreach among commuters and mobile populations.

This analysis should be interpreted in light of some limitations. Geocoding of residential locations for individuals diagnosed with TB at health facilities was based on patient-reported descriptions of locations and may have been biased towards specific landmarks or major intersections. As such, our findings should be interpreted as the potential yield of performing targeted case finding based on reported sites of residence in the absence of geocoded locations, rather than the yield of expanding contact investigation from actual sites of residence. Second, we used the prevalent TB diagnosed during door-to-door case finding to represent undiagnosed TB in the community in order to evaluate a simplified geographically targeted case finding approach, which does not account for the timing of screening relative to the initial individual’s diagnosis of TB at the health facility and does not explicitly address transmission risk. Instead, our work is an attempt to refine data-driven geographically targeted case finding to improve diagnostic yield at a lower cost than community-wide door-to-door testing. Finally, our study had limited statistical power despite testing nearly 8000 participants, reflecting the relatively low yield of TB cases in community-wide screening and the frequency of missing GPS coordinates in an implementation setting. In particular, we are not able to draw conclusions regarding the differences between individuals with prevalent TB living within 100 m and those living more than 100 m from an index household. However, the pragmatic nature of this study may inform TB programmes interested in exploring ways to expand the scope of contact investigation.

In conclusion, we found that in this densely populated Ugandan community, expanding household contact investigation to include screening within a 50-m radius of households affected by TB is at most moderately more efficient than screening the general population. These results suggest that screening other populations known to be at high risk for TB, including people living with comorbidities such as HIV and diabetes and individuals living in congregate settings, may be more efficient than spatially targeted screening centred on TB-affected households.

## Data Availability

Due to the sensitive nature of GPS coordinate data, requests for data sharing will be handled by the authors on case-by-case basis. The R code is available at https://github.com/korobsky/publications.
